# Physical Activity, Sports Practice, and Cognitive Functioning: The Current Research Status

**DOI:** 10.3389/fpsyg.2019.02658

**Published:** 2019-12-06

**Authors:** Antonio Hernández-Mendo, Rafael E. Reigal, Jeanette M. López-Walle, Sidonio Serpa, Oddrun Samdal, Verónica Morales-Sánchez, Rocío Juárez-Ruiz de Mier, José L. Tristán-Rodríguez, António F. Rosado, Coral Falco

**Affiliations:** ^1^Department of Social Psychology, Social Work, Anthropology and East Asian Studies, University of Málaga, Málaga, Spain; ^2^University of Málaga, Málaga, Spain; ^3^Autonomous University of Nuevo Leon, San Nicolás de los Garza, Mexico; ^4^Faculty of Human Motricity, University of Lisbon, Lisbon, Portugal; ^5^University of Bergen, Bergen, Norway; ^6^Department of Evolutionary Psychology and Education, University of Málaga, Málaga, Spain; ^7^Western Norway University of Applied Sciences, Bergen, Norway

**Keywords:** cognitive functioning, physical activity, physical-sports, brain, cognitive abilities

## Abstract

The evidence for the benefits of physical activity on cognitive functioning has increased in recent years. Although the relationship between these variables has been analyzed for decades, the development of evaluation techniques has resolved several issues and advanced this area of knowledge. Moreover, several authors have pointed out the association between the cognitive functioning of athletes and their performance in competition. These recent studies suggest that some specific cognitive abilities of athletes could help them become more effective and improve their chances of success. The objective of this paper was to identify the most relevant advances in these areas of study and to highlight more promising lines of research for the next few years. We have discussed findings from the application of different physical activity programs as well as the most significant cognitive performance variables for sports practice. The limitations of the findings were also discussed.

## Physical Activity, Sports, and the Brain

The relationship between physical exercise and cognitive functioning has received much attention in recent years (Northey et al., 2018; Moran et al., 2019). This has been an object of interest for decades, but much remains unknown. Neuroscience has advanced significantly, improving knowledge of brain functioning in response to different situations and its evolution over the course of people’s lives (Cabeza et al., 2018; Dumoulin et al., 2018). Scientists studying physical activity and sports have integrated this knowledge of brain functioning, using it to explain the contribution of physical exercise and how cognitive performance may increase performance in certain facets of sport (Fink et al., 2018; Hsu et al., 2018).

Techniques such as electroencephalography (Cheron et al., 2016; Gutmann et al., 2018), functional magnetic resonance imaging (Chaddock-Heyman et al., 2013; Fontes et al., 2015; Chen et al., 2016), positron emission tomography (Boecker and Drzezga, 2016), single photon emission tomography (Shih et al., 2019), or magnetoencephalography (Huang et al., 2016) have all improved the visualization and understanding of cognitive processes generated and developed in physical activity and sport contexts. The core of their contribution is the observation of brain changes during exercise, the impact of various tasks, and improvements in physical condition, which reflects this phenomenon (Becker et al., 2016; Jonasson et al., 2017; Schwarb et al., 2017).

In addition, research based on these techniques is complemented by other procedures developed in neurobiology and neurophysiology to explain changes in the brain that are attributable to physical exercise. Specifically, hypotheses such as neurogenesis, synaptogenesis, or angiogenesis and the action of biomolecules such as irisin, brain-derived neurotrophic factor (BDNF), insulin-like growth factor-1 (IGF-1), vascular endothelial growth factor (VEGF), cathepsin B or interleukin-6 (Tari et al., 2019) have been proposed. However, these theoretical foundations are still in the process of consolidation for humans. In addition, some of these hypotheses arouse controversy among researchers, such as neurogenesis in humans throughout the life cycle, though this should be resolved in the coming years (Sorrells et al., 2018; Lourenco et al., 2019; Pontifex et al., 2019; Voss et al., 2019).

In addition to an increased number of techniques available to assess the connection between physical exercise and brain functioning, other factors may have influenced the proliferation of this line of research. On the one hand, there have been warnings about increased sedentariness of lifestyles in some societies, prompting recommendations for physical activity to improve health among various sections of the population (Hynynen et al., 2016; Zylke and Bauchner, 2016). In this regard, it has been proposed that brain functioning would benefit from physical activity, allowing better development in childhood and adolescence or acting as a protector in aging processes (Erickson et al., 2015; Costigan et al., 2016). On the other hand, elite sport requires increased performance from athletes, thus encouraging the search for variables to improve the probability of success in competition. Cognitive functioning therefore has been an area of knowledge to which numerous researchers have aimed to contribute (Krenn et al., 2018; Sakamoto et al., 2018).

In professional sports, differences between athletes are sometimes very subtle. Although it is difficult to eliminate all uncertainty from their performance, technical staff and research groups have sought to analyze and identify variables influencing this outcome. Thus, it is unsurprising that there are innumerable studies of physical preparation, technical and tactical aspects, or psychological influences (Fister et al., 2018; Dalen et al., 2019; Henriksen et al., 2019). Among these studies, brain analysis has attracted intense attention in recent years, becoming a prolific field of study and application that will probably continue in future as technical resources are perfected.

## Physical Activity and Cognitive Functioning in Children, Adolescents, and the Elderly

### Childhood and Adolescence

Analysis of the benefits of physical activity on people is especially relevant in childhood and adolescence. These are stages when future lifestyles are established, and acquired habits have a very strong influence on the state of health throughout life. However, during childhood and adolescence the brain is under construction and requires appropriate learning processes. In addition, children and adolescents are in a phase when their personal and social development is conditioned by multiple changes, to which they must make efforts to adapt. Therefore, the possible benefits of the cerebral functioning that physical exercise could produce become essential elements for children’s and adolescents’ growth and integration into the environment (Wenner et al., 2013; Lubans et al., 2016). In fact, adequate cognitive development during early stages is thought to contribute to improvements in wellbeing and mental health in adulthood (Gale et al., 2012).

In recent years, multiple studies have highlighted significant associations between physical practice and abilities, such as attention and concentration, executive functions, cognitive functioning speed, memory, or language (e.g., Chaddock et al., 2011; Scudder et al., 2014; Donnelly et al., 2016; Li et al., 2017; Xue et al., 2019). Various investigations have analyzed the acute effects of physical exercise (Hillman et al., 2009b; Ellemberg and St-Louis-Deschênes, 2010; Chang et al., 2012a), the effects of a prolonged exercise program (Hillman et al., 2014; Reloba-Martínez et al., 2017), as well as correlations between cognitive functioning with people engaged in regular physical activity or physical fitness exercises (Hillman et al., 2005; Carson et al., 2016; Pérez-Lobato et al., 2016).

Numerous papers have studied the relationship between physical activity and cognitive functioning, highlighting the importance of physical fitness (Hillman et al., 2009a). That is, the effect of exercise on the brain is modulated by the overall impact of physical exertion on the body. Therefore, not only is physical exercise necessary, but it should have specific characteristics that improve study participants’ physical condition (Hötting and Röder, 2013; Reloba-Martínez et al., 2017; Reigal et al., 2019a). Among the manifestations of physical fitness, aerobic capacity best explains the association between physical exercise and cognitive development in children and adolescents, as several authors have highlighted (Pontifex et al., 2011; Herting et al., 2014).

Studies using neuroimaging techniques to explore these relationships have linked structural changes in the brain to exercise and the physical condition of children and adolescents. Authors such as Chaddock et al. (2010) have observed a higher volume in the hippocampus and the striated dorsal body with higher levels of aerobic fitness in children. Furthermore, Chaddock-Heyman et al. (2018) found an increase in the white matter microstructure of the genu of the corpus callosum after a 9-month program of moderate and vigorous physical activity in children. Likewise, in a group of obese children, Esteban-Cornejo et al. (2017) observed a relationship between cardiorespiratory capacity and speed/agility with the volume of gray matter in frontal, temporal, and subcortical regions, such as the premotor and supplementary motor cortex, the hippocampus, the caudate nucleus, as well as the inferior, superior, and parahippocampal temporal rotation.

### Elderly People

The relationship between physical exercise and brain functioning has also been analyzed with great intensity in elderly people. There is a decline in certain physical and cognitive capacities that compromise normal functioning and autonomy during this stage of life (Schiebener and Brand, 2017). In addition, events such as retirement, the appearance of age-related illnesses, or the reduction in social relationships due to the loss of loved ones may occur, leading to greater isolation. In recent years, a number of studies have suggested that physical exercise in elderly people has benefits for aspects of brain functioning, such as attention, memory, or executive functioning (Bherer et al., 2013; Kramer and Colcombe, 2018; Crespillo-Jurado et al., 2019). Regular moderate to vigorous physical exercise has been described as being protective against cognitive impairment and effective in maintaining adequate functioning in later life (Zhu et al., 2017). Therefore, promoting physical practice has become a potent strategy to improve elderly people’s adaptation to the environment, maintaining, or improving their state of health and increasing their quality of life.

It has been observed that improvements in aspects such as aerobic capacity, balance, strength, or body composition would reduce the impact of aging on the deterioration of the brain, cushioning its effects and maintaining mental skills for longer periods of time (Chang et al., 2012b; Kerr et al., 2013; Wilczyński et al., 2017). As with other populations, cognitive functioning in elderly people is associated with their physical condition and changes in the structure of the brain support their functional development (Reiter et al., 2015; Fernandes et al., 2017). For example, Erickson et al. (2011) observed that increases in aerobic capacity may be associated with improvements in spatial memory as well as increased BDNF concentration and volume in the hippocampus. Boyle et al. (2015) found that elderly people who were more active and had a lower body mass index had greater brain volume in areas such as the frontal and occipital lobes, in specific areas such as the frontal orbital cortex or the anterior cingulate gyrus, and had less dilation of cerebral ventricles.

In recent years, analyses of the impact of physical activity on neurodegenerative processes have received great attention. Several studies suggest that physical activity may be beneficial in response to conditions such as Alzheimer’s disease, both delaying its onset, mitigating its neuropathological effects, and improving brain functioning in patients (Phillips et al., 2015). Authors such as Lautenschlager et al. (2008) observed that, after a 24-week program of physical activity by elderly people, the rate of cognitive impairment in those at risk of dementia was reduced, suggesting that this type of intervention may inhibit the onset of the disease. It has also been observed that, in elderly people with a genetically increased risk of Alzheimer’s disease, improvements in aspect so the physical conditions, such as cardiorespiratory capacity, may mitigate the risk of Alzheimer’s disease (Tari et al., 2019). For this reason, it is considered that combined pharmacological treatments and more active lifestyles may be effective ways to counter this type of illness and other forms of dementia (Mortimer and Stern, 2019).

In addition, a large number of studies have indicated that physical activity may be useful in improving degenerative processes and symptoms in people with Parkinson’s disease (Fernández-del-Olmo et al., 2018). In this population, it has been observed that the regular practice of physical activity throughout life could protect against the emergence of the disease, and that greater quantity and intensity have protective effects against Parkinson’s disease (Paillard et al., 2015). Specifically, several authors have highlighted that aerobic exercise and cardiorespiratory capacity are positively related to brain processes, which again moderate the impact of Parkinson’s disease (Ahlskog, 2018).

### Limitations and Prospects for Future Research

Although many studies in recent years confirm the relationship between physical activity and cognitive functioning, there remains much to be understood. In addition, not all observed findings are supported by other studies, or they are based on small sample sizes with low statistical power. Thus, continued research is needed (Frederiksen et al., 2018). For greater precision in understanding the observed relationship between physical exercise and cognitive functioning and the possibilities for practical application, integration of knowledge from a range of scientific fields is recommended; these fields include sports science, neurobiology, neurophysiology, or neuropsychology as well as data from clinical and epidemiological studies. Furthermore, the transfer of findings from animal research to humans is required (Tari et al., 2019). According to recent research, there is some agreement on accepting the positive links observed between exercise and the brain, but the biological mechanisms underlying them require further examination (Fernández-del-Olmo et al., 2018).

Among the limitations of research to date is the heterogeneity of the studies and interventions, the lack of controls for strange variables, and small sample sizes. This has generated a collection of studies supporting the benefits of exercise on cognitive functioning, despite their moderate clinical value (Brasure et al., 2018). In addition, the exact amount and intensity of physical activity to meet individual needs has not yet been determined (Paillard et al., 2015). As an example, Frederiksen et al. (2018), studying a group of elderly Alzheimer’s patients, concluded that a 16-week (60 min/3 days per week) aerobic exercise program had no positive impact on cortical gray matter atrophy. However, disease symptoms improved and there were positive correlations in the intervention group between frontal cortical volume, exercise load, measures of attention, and cognitive processing speed. Thus, the authors suggested that longer interventions and a larger sample could generate more convincing results. The challenge therefore remains to find the most appropriate formulas for each case and thereby determine the optimal exercise program.

An attempt has been made to explain the benefits of physical activity for the brain and its functioning in multiple populations, both healthy and with some pathology. However, presumably the requirements for each age or disease are specific, in addition to the differential impact on each person (Phillips et al., 2015; Carson et al., 2016; Donnelly et al., 2016; Fernandes et al., 2017). For example, correlational or intervention studies in childhood and adolescence are conditioned by the natural development of these age groups and subject to various confounding factors, both intrapersonal and environmental, which impede the interpretation of the observed results (Chen et al., 2016; Chaddock-Heyman et al., 2018). Thus, one of the greatest challenges in coming years is to identify the appropriate load, intensity, volume, frequency, duration, and type of physical activity so that the effects on the development of the brain of the relevant population group will achieve the desired objectives. Therefore, extensive control of confounding variables is necessary to minimize their bias. This goal achieved, not only can physical exercise for health be recommended in general terms, but it can also be implemented in multiple specific educational and clinical programs to complement the actions recommended by other disciplines.

## Cognitive Functioning and High-Performance Sports

In the field of high-performance sports, the study of cognitive functioning has caught the attention of researchers. Vestberg et al. (2017) and Policastro et al. (2018) have shown that improved brain functioning in male and female athletes may enhance performance and predict success in competition. In general, it is assumed that this cognitive functioning may be more relevant in open sports requiring constant attention, management of multiple variables, or adaption to changing situations (Williams et al., 2011; Verburgh et al., 2014). Furthermore, good cognitive functioning may be a competitive advantage in disciplines with less variability but requiring high levels of concentration or attentional control (Memmert, 2009).

Studies have observed that better scores in executive functioning are related to greater expertise and success in football players (Verburgh et al., 2014; Huijgen et al., 2015). In turn, Roca et al. (2018) suggested that the creativity shown by adult football players in decision-making could be conditioned by their attentional capacity. In addition, Voss et al. (2010) pointed out that elite athletes tend to show better measures in aspects such as cognitive processing speed or various attentional tasks. Similarly, Wagner et al. (2014) concluded that cognitive aspects such as attentional capacity and executive functioning influence sports performance in handball. Moreover, Hänggi et al. (2015) pointed out that the brain structure can indicate the athlete’s functioning, making brain plasticity an interesting predictor of player behavior on the playing field.

Recently, following these findings and the need to find variables to explain athletes’ behaviors and success, the amount of research on how their brains work is increasing. Moreover, some researchers have attempted to train the athletes’ brains with the objective of modifying the way athletes respond to stimuli during games and improve their decisions (Calmels et al., 2004; Seo et al., 2012). Some of these studies have sought to modify the capacity of the athletes’ brains and have tried to improve their attention, processing speed, or different aspects of cognitive functioning in sports such as tennis, football, handball, basketball, badminton, rugby, or archery (Hagemann et al., 2006; Ducrocq et al., 2016; Romeas et al., 2016).

For cognitive assessment and training in sports, multiple strategies have been used, both classical paper and pencil or computerized tests (Memmert, 2009; Hernández-Mendo et al., 2012; Verburgh et al., 2014; Huijgen et al., 2015; Reigal et al., 2019b) as well as other more technologically advanced procedures (Memmert, 2009; Hernández-Mendo et al., 2012; Verburgh et al., 2014; Huijgen et al., 2015). In recent years, new electromechanical, digital, and combined devices have been developed, such as Fitlight Trainer^TM^, Dynavision D2^TM^, NeuroTracker^TM^, eye-tracking in sports, Vision Trainer^TM^, Senaptec Sensory Station, and Sports Vision Performance (M&S). Additionally, other products are based on technologies such as augmented or virtual reality. Overall, this new technology enabled notable progress in the assessment and cognitive preparation of athletes (Schack et al., 2014; Romeas et al., 2016; Appelbaum and Erickson, 2018). The rapid development of new technical measurement and training tools continuously increases the ecological validity of the measures by bringing the athlete’s training experience closer to an authentic game context. Therefore, the technology development opens up a very promising path for this line of research by offering possibilities that were previously unexplored.

### Limitations and Prospects for Future Research

Studies of the relationship between brain functioning and sports performance have evolved rapidly in recent decades following technological advances that have enabled the development of increasingly powerful instruments (Appelbaum and Erickson, 2018). This has created a series of opportunities for professionals as well as researchers to advance our understanding of the relationship between brain functioning and sports performance. Similarly, current limitations are likely to be reduced by the coming technologies. Among them are many laboratory-based procedures used to study how the brain works in sport contexts (Verburgh et al., 2014; Fink et al., 2018). This involves assessing the athletes’ behavior in artificial situations, which limits the ecological validity of these procedures. Although possibly quite accurate inferences may be drawn, there are variables inherent in the competitive context that are currently difficult to reproduce.

Therefore, to increase the usefulness of cognitive assessments and training in performance sports, it will be necessary to improve the transference of laboratory knowledge to real-life contexts. The available technology allows experiences to approach reality, but such strategies are limited by technologies that are still very invasive and difficult to use on the playing field (Romeas et al., 2016; Appelbaum and Erickson, 2018). As technical evolution makes it possible to evaluate and train brain function in sports environments, it will help to determine more precisely the most appropriate way to stimulate the brain to optimize sports performance.

Similarly, cognitive assessments of athletes must be adjusted to the requirements of their specific tasks. In other words, the needs of a defender in football are not the same as those of a striker, nor are the needs of a basketball player the same as those of a tennis player. Therefore, another challenge to be addressed more clearly is finding the right type of training for each athlete considering his/her sporting characteristics and goals (Vestberg et al., 2017). In this way, the usefulness of this type of training, as well as the demand from athletes and technical coaches, will be increased. Much progress has been made in recent decades in areas such as physical or technical–tactical training. Nevertheless, a core question remains: how should the brain be trained to optimize sports performance so that it benefits physical and psychological health? Hopefully this question will be clearly answered in the future.

## Author Contributions

AH-M, RR, JL-W, SS, OS, VM-S, RJ-R, JT-R, and CF participated in the design of the work and the bibliographic review, drafted the manuscript, and approved the final manuscript as submitted. All authors made substantial contributions to the final manuscript.

## Conflict of Interest

The authors declare that the research was conducted in the absence of any commercial or financial relationships that could be construed as a potential conflict of interest.
